# Effectiveness of a Smartphone App (e-12HR) in Improving Adherence to the Mediterranean Diet in Spanish University Students by Age, Gender, Field of Study, and Body Mass Index: A Randomized Controlled Trial

**DOI:** 10.3390/nu15071688

**Published:** 2023-03-30

**Authors:** Luis M. Béjar, Pedro Mesa-Rodríguez, Angélica Quintero-Flórez, María del Mar Ramírez-Alvarado, María Dolores García-Perea

**Affiliations:** 1 Department of Preventive Medicine and Public Health, School of Medicine, University of Seville, 41004 Seville, Spain; 2 Camas Health Center, 41900 Camas, Seville, Spain; 3 Department of Audiovisual Communication and Advertising, School of Communication, University of Seville, 41004 Seville, Spain; 4 Virgen Macarena University Hospital, 41009 Seville, Spain

**Keywords:** Mediterranean diet, dietary assessment, Mediterranean diet score, Mediterranean diet adherence, mobile applications, university students, food, population groups

## Abstract

There is an urgent need to implement intervention programs to promote adherence to the Mediterranean diet (AMD) in university students to prevent non-communicable diseases. A powerful tool for this is smartphone apps. Furthermore, it is necessary to determine the subgroups that are most likely to benefit from these technologies. The objective is to evaluate the effectiveness of an app (e-12HR) at improving AMD in a sample of Spanish university students and different strata. The study method was a controlled and randomized clinical trial over a four-week follow-up period and involving 385 participants (76.9% women). The participants were in two parallel groups: the control group (CG) and the intervention group (IG), with only the IG receiving feedback to improve their AMD. There were significant statistical improvements (with higher values in the IG) at week four, after no significant statistical differences at baseline (Week One): in the whole sample: +25.7% AMD index and +74.5% percentage with moderate/high AMD index. In the subgroups, seven of eight subgroups, ranging in AMD index from +17.8% (≥20 years) to +33.0% (<20 years); and for males, in weeks two (+27.9%) and three (+23.9%), but not at week four. In conclusion, e-12HR could improve AMD among university students (in the total sample and all subgroups, except ≥25 kg/m^2^).

## 1. Introduction

University students are particularly vulnerable to nutritional alterations mainly due to the fact that many are faced for the first time with the opportunity to make their own dietary choices [1,2], followed by the major changes they experience during their university years, both in terms of physical and social development [3]. In addition, university students are exposed to several factors, such as stress and lack of time, combined with scant information and an unfavorable food environment (accessibility or the attractiveness and price of certain food products and advertising), which make them less likely to maintain a healthy lifestyle [3,4].

Numerous epidemiological and nutritional studies have shown that a healthy diet, such as the Mediterranean diet (MD), improves the quality of life and helps prevent several non-communicable diseases (NCDs) such as cardiovascular diseases [5], obesity [6], cancer [7], and metabolic syndrome in adults [8]. The MD is a dietary pattern that is not only healthy but also sustainable: it is known for its low environmental impact, rich biodiversity, high sociocultural value, and benefits for local economies [9,10,11,12,13,14].

Although there is no single MD, common aspects of this healthy dietary pattern include a high intake of fruits, vegetables, legumes, and cereals; olive oil as the main fat; moderate amounts of dairy products and fish; and low amounts of meat and meat products [10,15,16]. Traditionally, Spain has followed an MD. However, cross-sectional studies have indicated that the eating habits of university students are moving away from Mediterranean dietary patterns towards unhealthy eating patterns [3,17,18].

The degree of adherence to MD (AMD) of Spanish university students has been the subject of several studies, which have shown that more than half of these students had poor AMD [3,17,18,19,20,21,22]. In general, the diets of Spanish university students presented unsatisfactory levels of consumption of fruit, vegetables, and legumes, with consumption of meat predominating over fish and with an excess of alcohol, fat and sugar [3,17,18,20,23,24].

Several studies have analyzed the AMD in different population strata of university students (by age, gender, field of study and body mass index (BMI)). According to age group, gender, and BMI, the results were inconsistent. With respect to age group, various studies have shown that AMD increased as age did [18,25], but another study found no difference with age group [21]. In terms of gender, one study shows that females had higher adherence than males [4], while another study found that females had poorer adherence than males [21]. Yet others find no differences according to gender [19]. With respect to BMI, certain studies have shown that AMD could have an impact on overweight/obesity [21,26], although this is not yet conclusive, as other authors have not found an association between AMD and BMI [27,28]. Finally, recent reports also show the possible difference in AMD depending on the degree or field of study; students of biomedical careers (i.e., medicine and nursing) showed higher levels of AMD than non-biomedical subjects [3,22]; when Health Science students were compared with those in other areas of study, it was observed that the latter had lower AMD [3,18,29]; higher adherence has been associated with more physically active students [23] and students whose subjects were linked to health-nutrition topics (e.g., Health Sciences or Biomedicine) [3]. Within the field of Health Sciences, no differences in AMD were observed between Medicine and Pharmacy students [21].

University students are an important target audience for public health actions, as this is a stage where they can consolidate previously learned patterns or learn new ones to replace old ones, ultimately establishing their nutritional habits for the future [1]. There is an urgent need to raise awareness among university students and implement intervention programs to promote better dietary habits and increase AMD as a measure to prevent the development of NCDs [18].

A powerful tool to promote and improve AMD among university students is the utilization of smartphone apps. Previously, the research team developed and validated a mobile app for measuring AMD called e-12HR [21,30]. This app has been updated and improved to include an automatic feedback process in which the app not only evaluates but also generates personalized comments for the user that contribute to the improvement of AMD [31]. The results of this study conducted among university students with the e-12HR application have been promising, suggesting relative effectiveness in the short term in improving the rate of AMD, as well as satisfactory usability of the app [31]. However, this previous study did not establish the effect of certain factors on the app’s ability to improve AMD, such as age, gender, field of study, or BMI.

As a continuation of the research team’s previous studies, the main hypothesis of this work was that the previously mentioned factors influencing AMD [3,18,20,21,25,26,29] could influence the ability of the e-12HR app to increase AMD. In the same vein, the main objective of this study was to evaluate the effectiveness of using the e-12HR app with feedback as compared to the app without feedback [31], at improving AMD in the whole sample of Spanish university students (this also included the identification of the food groups in which compliance with the MD recommendations had increased), and in different strata thereof. To the best of our knowledge, this is the first research to evaluate the influence of these factors (age, gender, field of study, and BMI) on the effectiveness of a smartphone application to improve AMD among university students. In addition, a secondary objective was to establish the usability of the app in the entire sample of Spanish university students.

## 2. Materials and Methods

### 2.1. Design Overview

The present study is a continuation of previous research and follows the same study protocol, which has been described by Béjar et al. [31] in detail (article free for readers published in *Nutrients* (an open-access journal), Volume October 2022). However, in the current study, the sample and the period for obtaining it were different. In brief, the present study is a randomized and controlled clinical trial with two parallel groups: the control group (CG) and the intervention group (IG). The study took place over a four-week period, and the trial registration can be found at ClinicalTrials.gov, NCT05532137.

### 2.2. Setting and Participants

The Faculties of Medicine, Pharmacy and Communication at the University of Seville (Andalusia, Spain, South of Europe) were included in the study.

Confidentiality was guaranteed in accordance with the Organic Law on the Protection of Personal Data and the Law 14/2007 on Spanish biomedical research.

Inclusion criteria: Both genders, over 18 years old, students of Medicine, Pharmacy and Communication (University of Seville) and possess a smartphone.

Exclusion criteria: intolerance to any food, chronic diseases such as diabetes or pregnancy (situations which may require specific dietary recommendations).

A member of the research group explained the study to potential participants, including objectives, risks and benefits of the research; e-12HR application functionalities; and how to participate (the students had to send an e-mail to the research team).

When the research team received an e-mail from a student, they replied with another e-mail including the following documents: Document one: informed consent to be signed by the student and returned by e-mail; Document two: personal data (sex, date of birth, Faculty, weight, height, and smoking status) to be completed by the student and returned by e-mail; Document three: personal code (with numbers and letters); Document four: instructions for downloading e-12HR (a free app available on the App Store or Play Store); Document five: information for using e-12HR app; and Document six: information about the characteristics of the MD.

This protocol was selected to obtain a high participation rate, avoid unnecessary travel to complete or sign documents, as well as to avoid wasting paper.

Recruitment of participants: September–October 2022.

The students who successfully completed the protocol were entered into the raffle for school materials (valued at EUR 500).

### 2.3. Randomization and Masking

Four random classrooms were selected in each school (Medicine, Pharmacy and Communication): twelve classes in all. Of the four selected classrooms in each school, two were assigned to each group (CG and IG), according to the following sequence: first class: IG; second class: CG; third class: IG; and fourth class: CG. In this way, all students in the same class were assigned to the CG or the IG by probability single-stage cluster sampling.

Due to the nature of the study, the students could not be blinded. However, the statistical analysis of the data was performed by a person who remained blinded throughout the study. In addition, each participant only had access to one version of the application (CG: ‘non-feedback’ e-12HR version; IG; ‘feedback’ e-12HR version). This was possible by assigning personal alphanumeric codes (mentioned in the previous section).

**Figure 1:**
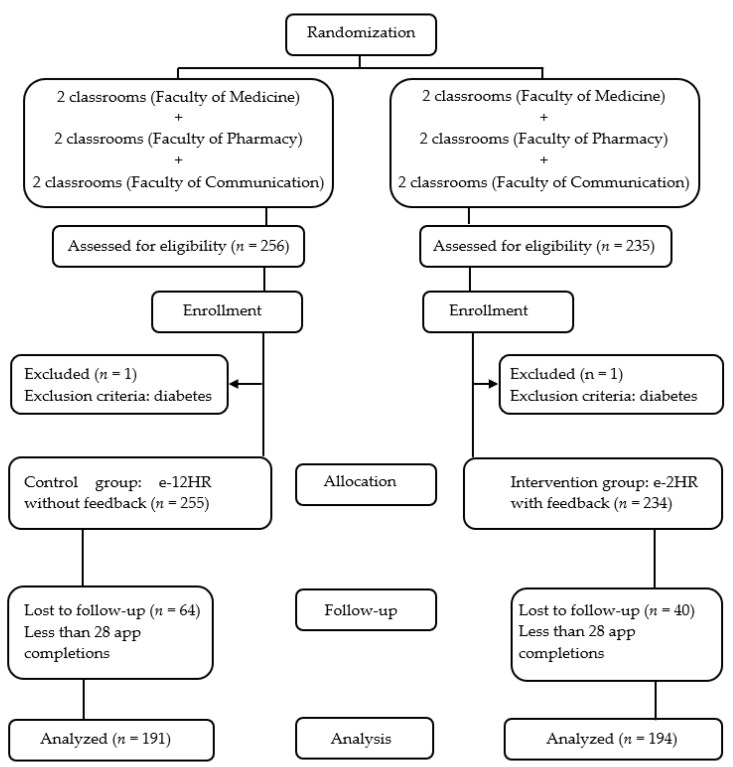
Flowchart of the Study.

The allocation sequence is detailed in Figure 1.

### 2.4. Intervention

CG: ‘non-feedback’ e-12HR version.

IG: ‘feedback’ e-12HR version.

The structure and functions of the e-12HR application (‘non-feedback’ e-12HR and ‘feedback’ e-12HR versions) have been described, in detail, by Béjar et al. (in “e-12HR App” in the Section 2.4) [31]. No changes were made in the application compared to the original study protocol. In brief, the ‘non-feedback’ e-12HR version allowed the user to collect food consumption data; the ‘feedback’ e-12HR version allowed for the collection of food consumption data and, additionally, issued personalized recommendations to improve AMD.

### 2.5. Follow-Up and Outcome Measures

In order to assess the effect of the app (‘feedback’ e-12HR), follow-up was carried out at week one (baseline), week two, week three and week four of monitoring.

Main result variable: the change in the AMD score at weeks two, three and four of monitoring.

Secondary result variables: the personal information variables, and the answers to the usability rating questionnaire for e-12HR (see Section 2.7).

#### Adherence to the MD

Every week during the four-week study period, the AMD index score (specifically, Mediterranean Diet Serving Score (MDSS) index [32]) was calculated manually (for CG and IG). Calculation of the AMD index has been described, in detail, by Béjar et al. (in “AMD Assessment”, in the Section 2.4) [31].

### 2.6. Sample Size Calculation

The sample size estimation was made for the main result variable: the change in the MDSS index. Considering a standard deviation of 2.7 points in the MDSS index and a dropout rate of 20.6% (from a previous study using e-12HR [31]), α = 0.05 and β = 0.20, bilateral test, 292 participants (146 per group) were needed to detect a variation of 1 point in MDSS index in the IG versus CG.

The sample size was calculated using the nQuery Advisor Release 7.0 program.

### 2.7. Usability Rating Questionnaire for e-12HR

After the four-week monitoring period, a member of the research group sent e-mails to students (with a usability rating questionnaire for the e-12HR app [31], included in Appendix A, Table A1).

### 2.8. Ethical Considerations

Participants were required to sign the informed consent prior to inclusion in the study, according to the Declaration of Helsinki.

The study was approved by the University of Seville’s Research Ethics Committee on 30 March 2022 (internal identifier: 2813-N-21). Trial Registration: ClinicalTrials.gov, identifier NCT05532137.

### 2.9. Statistical Analysis

The results were displayed as numbers (percentages) for qualitative variables and as means (standard deviations) for quantitative variables.

The data were tested for normality using the nonparametric Kolmogorov–Smirnov test.

In order to compare proportions, the chi-square test (or Fisher exact test) was carried out as appropriate, and Student’s *t*-test (or the nonparametric Mann–Whitney U-test) was used for the comparison of continuous variables.

A *p*-value < 0.05 was considered significant.

Statistical analyses: using the SPSS statistical software package version 26.0 (SPSS Inc., Chicago, IL, USA).

## 3. Results

### 3.1. Sample and Adherence to the Study

A total of 491 students signed the informed consent forms; however, two students were excluded for being diabetic (one student from the IG and one from the CG), failing to meet the selection criteria. Of those who signed, 104 (64 in the CG and 40 in the IG) were considered to be non-responsive, as they did not complete the study’s 28-day follow-up period (Figure 1). The data for these individuals were not included in the later statistical analysis.

Overall, the study response rate was 78.7% (385/489)–74.9% (191/255) in the CG, and 82.9% (194/234) in the IG- (Figure 1). According to faculties, the study response rates were: Medicine: 77.3% (153/198)–74.2% (66/89) in the CG, and 79.8% (87/109) in the IG. Pharmacy: 83.8% (140/167)–80.0% (68/85) in the CG, and 87.8% (72/82) in the IG. Communication: 74.2% (92/124)–70.4% (57/81) in the CG, and 81.4% (35/43) in the IG.

### 3.2. Personal Information of the Participants

Table 1 shows the personal information of the participants (CG and IG).

No significant statistical differences were observed in the variables studied (CG versus IG) except in the “field of study”, although, in both groups, Health Science students exceeded 70% of the sample (Table 1).

No significant statistical differences were observed in the variables studied between responsive (those who completed the study) and non-responsive (those who did not) participants.

The responsive participants registered their daily consumption for the 19 food groups included in the study (fruits, vegetables, cereals -breakfast cereals, pasta, rice, and bread-, olive oil, milk and dairy products, nuts, fermented beverages -wine and beer-, potatoes, legumes, eggs, fish, white meat, red meat, processed meats and sweets [31,32]) for 10,780 days altogether (385 participants and a 28-day follow-up period). This value represents a collected total of 204,820 data points on daily consumption for the food groups.

### 3.3. MDSS Index

For both groups (CG and IG), scoring for the MDSS was calculated manually by the research team [31]. In this process, the research team modified the obvious errors made by participants during data entry (as it was considered that the data must have been introduced as milliliters or grams instead of standard servings). For example, on several occasions, a value between 50 and 70 was introduced for the question, “How many servings of pasta have you consumed today?” The research team considered that these values indicated a consumption between 50 and 70 g, which is the equivalent of one serving. In any case, only 692 data points were modified by the research team (out of a total of 204,820 registered data points: 0.31%).

### 3.4. Effect of the Intervention

#### 3.4.1. Effect of the Intervention in Terms of Variation in MDSS Index and Percentage of Participants with Moderate/High (≥9) MDSS Index in the Whole Study Sample

There were significant statistical differences for both the MDSS index and the percentage of participants with moderate/high (≥9) MDSS index (CG versus IG) in weeks two, three and four, with higher values in the IG (no significant differences in week one): for the MDSS index with 1.25, 1.78 and 1.93 points of improvement, respectively, and for the percentage of participants with moderate/high (≥9) MDSS index with increases of 19.2, 20.7 and 24.2 percentage points, respectively (Table 2).

#### 3.4.2. Effect of the Intervention in Terms of Variation in Food Groups in the Whole Study Sample

Table 3 shows the percentage of participants that meet the consumption criteria for each food group, for the CG and the IG, throughout the four weeks of follow-up in the whole study sample. In addition, Table 3 shows the MDSS index throughout the four weeks of follow-up (CG and IG) in the whole study sample (these data have already been previously collected in Table 2; however, they have been included again for easy comparison by readers with the data from food groups).

No statistically significant differences were observed at week one in any of the food groups except fish. It should be noted that all food groups that, according to the MDSS index, present a daily consumption recommendation [32] (except fermented beverages) showed statistically significant differences (CG versus IG) throughout the four weeks of follow-up: for fruits, cereals, olive oil and nuts in weeks two, three and four; for vegetables in weeks three and four; and for milk and dairy products in week four. Additionally, regarding the food groups with weekly recommendations [32], there was a statistically significant difference (CG versus IG) for legumes at week four. In those subgroups in which statistically significant differences were observed at week four (and no significant differences at week one), CG versus IG, the IG showed higher percentages, specifically: +14.7 percentage points for fruits, +11.2 percentage points for vegetables, +17.2 percentage points for cereals, +15.7 percentage points for olive oil, +10.8 percentage points for milk and dairy products, +7.1 percentage points for nuts and, finally, +10.4 percentage points for legumes (Table 3).

#### 3.4.3. Effect of the Intervention in Terms of Variation in MDSS Index in Different Subgroups of the Study Sample

The differences were statistically significant considering the MDSS index (CG versus IG): in weeks two, three and four for the subgroups <20 years, female, Health Science and <25 kg/m^2^; in weeks two and three for male; and in week four for ≥20 years and Non-Health Science. However, there were no statistically significant differences at any week in the subgroup ≥25 kg/m^2^. In those subgroups in which statistically significant differences were observed at week four (no significant differences at week one in any of the subgroups), CG versus IG, the IG showed higher MDSS index values, specifically: +2.34 points for <20 years, +1.43 points for ≥20 years, +2.03 points for female, +1.86 points for Health Science, +1.39 points for Non-Health Science, and, finally, +2.18 for <25 kg/m^2^ (Table 4).

### 3.5. Usability Rating Questionnaire for e-12HR

This questionnaire was answered by 127 students (66 from the CG and 61 from the IG).

The responses of the users are shown in Table 5.

Considering the 127 participants who answered the questionnaire, all (100%) reported that e-12HR was easy to complete, and most of them indicated that: (1) the application contained understandable questions (97.0% CG and 91.8% IG); (2) the app contained understandable feedback (only for the IG, 85.2%); (3) they would be willing to complete the e-12HR app again, (53.0% CG and 63.9% IG); and (4) the time to complete the task was 2 min or less (56.1% CG and 57.4% IG). There were no statistically significant differences (CG versus IG) for any of the questions on the questionnaire (Table 5).

## 4. Discussion

Few randomized and controlled clinical trials have analyzed the effectiveness of promoting the MD (in Spanish adults) of smartphone apps with certain similarities to e-12HR [33,34,35,36]. The smartphone applications EVIDENT II [33,34,35] and SalBi Educa Nutrition [36] shared functionalities with e-12HR; all of them allowed entry of food intake and offered personalized dietary advice. According to Recio-Rodríguez et al. [34], future research should clarify the possible effects certain factors (such as age, gender, or educational level) might have on the success of using diet applications; in this way, it would be possible to determine the subgroups that are most likely to benefit from the support of these technologies. This work is pioneering since, to the best knowledge of the research team, it was the first to evaluate the influence of certain factors (such as age, gender, field of study, and BMI) on the ability of an application to improve AMD in adults (specifically, university students).

The main findings of this work were (significant intergroup statistical modifications): First: in relation to the main objective, in the entire sample of Spanish university students, the increase in AMD from week two throughout the follow-up of the study (at week four the increase was favorable to IG versus CG by +25.7% for MDSS index and by +74.5% for the percentage of participants with moderate/high (≥9) MDSS index) (Table 2); with improvements observed in all food groups with daily consumption recommendations [32] (except fermented beverages), as well as for legumes (Table 3). In the subgroups, the increase for MDSS index throughout the follow-up of the study in seven of the eight subgroups considered (at week four, the increase was favorable to IG versus CG by +33.0% for <20 years, +17.8% for ≥20 years, +27.1% for female, +23.5% for Health Science, +21.0% for Non-Health Science, and, finally, +28.8% for <25 kg/m^2^); for male, there were higher data of MDSS index in IG versus CG in weeks two (+27.9%) and three (+23.9%), but not at week four (Table 4). Second: regarding the secondary objective, the responses of the participants to the usability rating questionnaire for e-12HR have shown that the app has good usability in the whole of the sample and the different strata considered (Table 5). Usability is an important aspect in the field of applications. In fact, for healthcare professionals, the three principal criteria for selecting a “Nutrition and Diet” app for their clients/patients were [37]: ease of use (e-12HR obtained very positive responses to the usability rating questionnaire), apps being free of charge (download of e-12HR is free) and validated (e-12HR is a pre-validated app [30,38,39,40,41]).

The increase in MDSS index (CG versus IG) with the use of e-12HR can be considered moderate (with higher values in the IG): in the entire sample of university students, +25.7%; in the subgroups, ranging from +17.8% (for ≥20 years) to +33.0% (for <20 years). However, this moderate increase among university students could be considered, at the same time, promising. University students are not characterized by being in high motivation stages to change a lifestyle factor (preparation or action, following the model of Prochaska and Velicer [42]), i.e., they are, in general, participants with little motivation for diet change. In this study, the motivation stage for the change of the participants was not formally collected; however, in the informal contact with the students, they stated that, for the most part, they were not in phases of high motivation. Therefore, the research team hypothesizes that the use of e-12HR could lead to greater increases in AMD with a sample of participants with greater motivation to change their diet (new studies will be performed to test this hypothesis, see “Future Research Related to the Current Study” Section).

As previously mentioned, some randomized and controlled clinical trials have used applications with certain similarities to e-12HR to improve AMD in Spanish adults [33,34,35,36]; however, these studies differed from this study and other ones by the research team [31] (both carried out among university students, using MDSS index and with a four-week follow-up period), in the participants selected (patients of healthcare centers [33,34,36] or patients with diabetes mellitus type 2 [35]), the length of the follow-up period (three months [33,34,35], only the study by Gonzalez-Ramirez et al. [36] had a similar duration of four weeks), and the AMD index used (Mediterranean Diet Adherence Screener (MEDAS) [33,34,35,36]). These differences between studies should be considered when comparing the results. In the intergroup comparisons (CG versus IG), in line with the results of this work and those of a previous study by the research team [31] (both with similar results in the entire sample -previous study: +17.4% for MDSS index and +61.9% for the percentage of participants with moderate/high (≥9) MDSS index for the IG-), statistically significant differences were observed (with higher values in the IG) in the study by Alonso-Domínguez et al. [35]: with moderate increases in MEDAS index and percentage of participants with adequate AMD (MEDAS score ≥ 9 points) and, according to food groups, with improvements in the consumption of several food groups, such as olive oil, vegetables, fruits, fish, commercial baked, nuts, sofrito sauce, and white meats after three months. It should be noted that e-12HR’s effect was noticeable after only four weeks of the intervention. It must be considered that, in the study by Alonso-Domínguez et al. [35], the intervention combined a food workshop, five walks and a smartphone application (EVIDENT II). Due to the multifactorial nature of the intervention, it is not possible to know which component produced the change in the IG in comparison to the CG [35]. No intergroup modifications (CG versus IG) were shown in the rest of the studies (for the MEDAS index, for the percentage of participants with adequate AMD MEDAS score ≥ 9 points, or for food groups) throughout the periods of use of the applications [33,34,36]. However, this should not be interpreted as these applications not being effective at improving the quality of the diet; in fact, EVIDENT II and SalBi Educa Nutrition apps proved to be useful for significantly increasing carbohydrate intake and decreasing total fat intake (CG versus IG, with higher values in the IG) [34,36]. Intakes of macro and micronutrients have not been measured in the current study.

This study presents several limitations. First, all the data collected were self-administered: the participants completed the daily nutrition questionnaire using the app (e-12HR being a self-reporting method, it presents the inherent limitations of this type of tool, described in detail in the bibliography [43,44,45,46,47,48,49]), and, at the end of the study, answered the usability rating questionnaire for e-12HR. In addition, the intervention was short (four weeks), and the long-term AMD index (once the use of the app has finished) is unknown. Furthermore, there was a relatively small number of individuals in some of the subgroups analyzed: male (*n* = 89), Non-Health Science (*n* = 92), and ≥25 kg/m^2^ (*n* = 60). Finally, the nature of this study made it impossible to blind the participants or to guarantee that the participants were not using another nutrition app during the follow-up period. Regarding the latter, a multifactorial intervention (combining the simultaneous use of e-12HR and another application to improve AMD) would mean that it would be impossible to know which component produced the change in the IG.

### Future Research Related to the Current Study

In those subgroups of the population that have improved AMD with the use of e-12HR, the research team intends to evaluate the possible increase in the MDSS index by combining the use of the app with counseling (counseling focused on food groups that have not improved consumption with the use of e-12HR, such as eggs, white meat, red meat, etc.): CG (‘non-feedback’ e-12HR version) versus IG (‘feedback’ e-12HR version + counseling) [35]. Additionally, the research team intends to evaluate the effectiveness of e-12HR at improving AMD among participants truly motivated to change their diet (i.e., who are in the preparation or action stages of change [42]).

## 5. Conclusions

The results of this study support recommending the use of e-12HR in university students as a tool to improve AMD in the short term, in the total sample and in all its subgroups, except ≥25 kg/m^2^ (group in which no improvement was observed in AMD throughout the follow-up period). Additionally, the application presents satisfactory usability in the whole of the sample and the different strata considered.

## Figures and Tables

**Table 1:** Characteristics of the Participants in the Study.

	All		Control Group		Intervention Group		*p*-Value
Characteristics	*n* (%)	Mean (SD)	*n* (%)	Mean (SD)	*n* (%)	Mean (SD)	
Participants who Completed the Study	385 (100)	- *	191 (100)	-	194 (100)	-	-
Age (Years)	-	20.7 (3.8)	-	20.3 (3.8)	-	21.0 (3.8)	0.357 **
<20	202 (52.5)	-	103 (53.9)	-	99 (51.0)	-	0.569 ***
≥20	183 (47.5)	-	88 (46.1)	-	95 (49.0)	-	
Gender							
Females	296 (76.9)	-	142 (74.3)	-	154 (79.4)	-	0.241 ***
Males	89 (23.1)	-	49 (25.7)	-	40 (20.6)	-	
Field of Study							
Health Science (Medicine + Pharmacy)	293 (76.1)	-	134 (70.2)	-	159 (82.0)	-	0.007 ***
Non-Health Science (Communication)	92 (23.9)	-	57 (29.8)	-	35 (18.0)	-	
BMI (kg/m^2^)	-	22.2 (3.5)	-	22.0 (3.3)	-	22.3 (3.6)	0.630 **
<25	325 (84.4)	-	163 (85.3)	-	162 (83.5)	-	0.620 ***
≥25	60 (15.6)	-	28 (14.7)	-	32 (16.5)	-	
Smoking Status							
No	341 (88.6)	-	166 (86.9)	-	175 (90.2)	-	0.310 ***
Yes	44 (11.4)	-	25 (13.1)	-	19 (9.8)	-	
Physical Activity Status (Minutes/Week)							
≥150	249 (64.7)	-	122 (63.9)	-	127 (65.5)	-	0.744 ***
<150	136 (35.3)	-	69 (36.1)	-	67 (34.5)	-	

* Not applicable. SD: standard deviation. BMI: body mass index. *p*-value: differences between control group versus intervention group, ** Mann–Whitney U-test, and *** chi-square test. A *p*-value < 0.05 is considered significant.

**Table 2:** Mediterranean Diet Serving Score (MDSS) Index and Percentage of Participants with Moderate/High (≥9) MDSS Index Throughout the Four Weeks of Follow-Up in the Whole Study Sample.

	Week Number			
MDSS Index	Week 1	Week 2	Week 3	Week 4
Control Group, Mean (SD)	7.10 (2.55)	7.49 (2.59)	7.46 (2.62)	7.52 (2.71)
Intervention Group, Mean (SD)	7.61 (2.79)	8.74 (3.08)	9.24 (3.53)	9.45 (3.40)
*p*-value	0.064	0.000	0.000	0.000
Percentage of Participants with Moderate/High (≥9) MDSS Index	Week 1	Week 2	Week 3	Week 4
Control Group, *n* (%)	50 (26.2)	56 (29.3)	60 (31.4)	62 (32.5)
Intervention Group, *n* (%)	66 (34.0)	94 (48.5)	101 (52.1)	110 (56.7)
*p*-value	0.094	0.000	0.000	0.000

*p*-value: differences control group versus the intervention group in each of the four study weeks, Mann–Whitney U-test (MDSS index) and chi-square test (percentage). A *p*-value < 0.05 is considered significant.

**Table 3:** Percentage of Participants that Meet the Consumption Criteria of Mediterranean Diet Serving Score (MDSS) Index for Each Food Group Throughout the Four Weeks of Follow-Up in the Whole Study Sample.

Criteria MDSS for Food Group	Week Number			
Fruits (1–6 Serving/Day)	Week 1	Week 2	Week 3	Week 4
Control Group, *n* (%)	88 (46.1)	82 (42.9)	87 (45.5)	88 (46.1)
Intervention Group, *n* (%)	94 (48.5)	104 (53.6)	114 (58.8)	118 (60.8)
*p*-value	0.640 *	0.036 *	0.009 *	0.004 *
Vegetables (≥2 Serving/Day)	Week 1	Week 2	Week 3	Week 4
Control Group, *n* (%)	13 (6.8)	18 (9.4)	20 (10.5)	18 (9.4)
Intervention Group, *n* (%)	13 (6.7)	29 (14.9)	35 (18.0)	40 (20.6)
*p*-value	0.967 *	0.098 *	0.034 *	0.002 *
Cereals (1–6 Serving/Day)	Week 1	Week 2	Week 3	Week 4
Control Group, *n* (%)	83 (43.5)	94 (49.2)	90 (47.1)	96 (50.3)
Intervention Group, *n* (%)	74 (38.1)	124 (63.9)	120 (61.9)	131 (67.5)
*p*-value	0.289 *	0.004 *	0.004 *	0.001 *
Olive oil (1–4 Serving/Day)	Week 1	Week 2	Week 3	Week 4
Control Group, *n* (%)	101 (52.9)	99 (51.8)	98 (51.3)	98 (51.3)
Intervention Group, *n* (%)	102 (52.6)	122 (62.9)	126 (64.9)	130 (67.0)
*p*-value	0.953 *	0.028 *	0.007 *	0.002 *
Milk and Dairy Products (1–3 Serving/Day)	Week 1	Week 2	Week 3	Week 4
Control Group, *n* (%)	129 (67.5)	132 (69.1)	126 (66.0)	126 (66.0)
Intervention Group, *n* (%)	146 (75.3)	148 (76.3)	145 (74.7)	149 (76.8)
*p*-value	0.094 *	0.114 *	0.059 *	0.019 *
Nuts (1–2 Serving/Day)	Week 1	Week 2	Week 3	Week 4
Control Group, *n* (%)	11 (5.8)	7 (3.7)	10 (5.2)	13 (6.8)
Intervention Group, *n* (%)	21 (10.8)	27 (13.9)	29 (14.9)	27 (13.9)
*p*-value	0.072 *	0.000 *	0.002 *	0.022 *
Fermented Beverages (0–2 Serving/Day)	Week 1	Week 2	Week 3	Week 4
Control Group, *n* (%)	186 (97.4)	185 (96.9)	183 (95.8)	186 (97.4)
Intervention Group, *n* (%)	188 (96.9)	188 (96.9)	193 (99.5)	190 (97.9)
*p*-value	0.780 *	0.978 *	0.019 **	0.749 **
Potatoes (≤3 Serving/Week)	Week 1	Week 2	Week 3	Week 4
Control Group, *n* (%)	99 (51.8)	100 (52.4)	98 (51.3)	96 (50.3)
Intervention Group, *n* (%)	98 (50.5)	88 (45.4)	85 (43.8)	93 (47.9)
*p*-value	0.796 *	0.170 *	0.141 *	0.648 *
Legumes (≥2 Serving/Week)	Week 1	Week 2	Week 3	Week 4
Control Group, *n* (%)	58 (30.4)	70 (36.6)	68 (35.6)	61 (31.9)
Intervention Group, *n* (%)	73 (37.6)	78 (40.2)	82 (42.3)	82 (42.3)
*p*-value	0.133 *	0.473 *	0.180 *	0.036 *
Eggs (2–4 Serving/Week)	Week 1	Week 2	Week 3	Week 4
Control Group, *n* (%)	89 (46.6)	90 (47.1)	90 (47.1)	88 (46.1)
Intervention Group, *n* (%)	83 (42.8)	84 (43.3)	87 (44.8)	91 (46.9)
*p*-value	0.452 *	0.451 *	0.654 *	0.870 *
Fish (≥2 Serving/Week)	Week 1	Week 2	Week 3	Week 4
Control Group, *n* (%)	83 (43.5)	88 (46.1)	90 (47.1)	82 (42.9)
Intervention Group, *n* (%)	109 (56.2)	113 (58.2)	124 (63.9)	116 (59.8)
*p*-value	0.012 *	0.017 *	0.001 *	0.001 *
White Meat (2–3 Serving/Week)	Week 1	Week 2	Week 3	Week 4
Control Group, *n* (%)	43 (22.5)	62 (32.5)	49 (25.7)	53 (27.7)
Intervention Group, *n* (%)	49 (25.3)	43 (22.2)	51 (26.3)	45 (23.2)
*p*-value	0.528 *	0.023 *	0.887 *	0.305 *
Red/processed Meat (<2 Serving/Week)	Week 1	Week 2	Week 3	Week 4
Control Group, *n* (%)	14 (7.3)	23 (12.0)	27 (14.1)	23 (12.0)
Intervention Group, *n* (%)	25 (12.9)	28 (14.4)	34 (17.5)	34 (17.5)
*p*-value	0.071 *	0.489 *	0.362 *	0.130 *
Sweets (≤2 Serving/Week)	Week 1	Week 2	Week 3	Week 4
Control Group, *n* (%)	65 (34.0)	68 (35.6)	66 (34.6)	68 (35.6)
Intervention Group, *n* (%)	64 (33.0)	83 (42.8)	91 (46.9)	86 (44.3)
*p*-value	0.829 *	0.149 *	0.014 *	0.081 *
MDSS index	Week Number			
Considering All Food Groups	Week 1	Week 2	Week 3	Week 4
Control Group, Mean (SD)	7.10 (2.55)	7.49 (2.59)	7.46 (2.62)	7.52 (2.71)
Intervention Group, Mean (SD)	7.61 (2.79)	8.74 (3.08)	9.24 (3.53)	9.45 (3.40)
*p*-value	0.064 ***	0.000 ***	0.000 ***	0.000 ***

SD: standard deviation. *p*-value: differences in the control group versus the intervention group in each of the four study weeks, * chi-square test, ** Fisher exact test and *** Mann–Whitney U-test. A *p*-value < 0.05 is considered significant.

**Table 4:** Mediterranean Diet Serving Score (MDSS) Index Throughout the four Weeks of Follow-Up in Different Subgroups of the Study Sample (by age, gender, field of study, and body mass index).

MDSS Index				
Subgroups by Age (Years)	Week Number			
<20	Week 1	Week 2	Week 3	Week 4
Control Group, Mean (SD)	6.78 (2.46)	6.93 (2.43)	7.00 (2.64)	7.08 (2.64)
Intervention Group, Mean (SD)	7.56 (3.00)	8.82 (3.41)	9.52 (3,73)	9.42 (3.37)
*p*-value	0.095 *	0.000 *	0.000 *	0.000 *
≥20	Week 1	Week 2	Week 3	Week 4
Control Group, Mean (SD)	7.48 (2.61)	8.15 (2.63)	8.00 (2.50)	8.05 (2.71)
Intervention Group, Mean (SD)	7.67 (2.56)	8.65 (2.70)	8.95 (3.31)	9.48 (3.46)
*p*-value	0.436 *	0.158 *	0.073 *	0.002 **
Subgroups by Gender	Week Number			
Female	Week 1	Week 2	Week 3	Week 4
Control Group, Mean (SD)	7.18 (2.53)	7.52 (2.70)	7.49 (2.71)	7.50 (2.75)
Intervention Group, Mean (SD)	7.53 (2.69)	8.55 (2.95)	9.27 (3.45)	9.53 (3.20)
*p*-value	0.286 *	0.002 *	0.000 *	0.000 *
Male	Week 1	Week 2	Week 3	Week 4
Control Group, Mean (SD)	6.88 (2.61)	7.41 (2.26)	7.37 (2.37)	7.59 (2.63)
Intervention Group, Mean (SD)	7.95 (3.15)	9.48 (3.48)	9.13 (3.88)	9.15 (4.12)
*p*-value	0.082 *	0.002 **	0.015 **	0.109 *
Subgroups by Field of Study	Week Number			
Health Science	Week 1	Week 2	Week 3	Week 4
Control Group, Mean (SD)	7.25 (2.55)	7.82 (2.69)	7.79 (2.70)	7.91 (2.69)
Intervention Group, Mean (SD)	7.88 (2.83)	9.07 (3.21)	9.63 (3.57)	9.77 (3.44)
*p*-value	0.067 *	0.001 *	0.000 *	0.000 *
Non-Health Science	Week 1	Week 2	Week 3	Week 4
Control Group, Mean (SD)	6.74 (2.53)	6.72 (2.16)	6.68 (2.25)	6.61 (2.56)
Intervention Group, Mean (SD)	6.40 (2.28)	7.23 (1.73)	7.46 (2.74)	8.00 (2.82)
*p*-value	0.779 *	0.105 *	0.207 *	0.016 *
Subgroups by BMI (kg/m^2^)	Week Number			
<25	Week 1	Week 2	Week 3	Week 4
Control Group, Mean (SD)	7.21 (2.59)	7.56 (2.69)	7.58 (2.73)	7.58 (2.76)
Intervention Group, Mean (SD)	7.77 (2.89)	8.90 (3.14)	9.60 (3.62)	9.76 (3.44)
*p*-value	0.084 *	0.000 *	0.000 *	0.000 *
≥25	Week 1	Week 2	Week 3	Week 4
Control Group, Mean (SD)	6.46 (2.22)	7.11 (1.89)	6.79 (1.79)	7.18 (2.43)
Intervention Group, Mean (SD)	6.81 (2.09)	7.94 (2.61)	7.38 (2.34)	7.91 (2.75)
*p*-value	0.450 *	0.161 **	0.275 **	0.286 **

SD: standard deviation. BMI: body mass index. *p*-value: differences control group versus the intervention group in each of the four study weeks, * Mann–Whitney U-test and ** Student’s *t*-test. A *p*-value < 0.05 is considered significant.

**Table 5:** Responses to the Usability Rating Questionnaire for e-12HR.

All (127)	
Questions	Answers
1. Easy to Complete	Strongly Agree + Agree
Control Group, *n* (%)	66 (100)
Intervention Group, *n* (%)	61 (100)
*p*-value	-
2. Understandable Questions	Strongly Agree + Agree
Control Group, *n* (%)	64 (97.0)
Intervention Group, *n* (%)	56 (91.8)
*p*-value	0.259 ***
3. Understandable Feedback (Only for Intervention Group)	Strongly Agree + Agree
Control Group, *n* (%)	-
Intervention Group, *n* (%)	52 (85.2)
*p*-value	-
4. I Would Be Willing to Complete Again	Strongly Agree + Agree
Control Group, *n* (%)	35 (53.0)
Intervention Group, *n* (%)	39 (63.9)
*p*-value	0.213 **
5. Time to Complete	≤2 Minutes/Day
Control Group, *n* (%)	37 (56.1)
Intervention Group, *n* (%)	35 (57.4)
*p*-value	0.881 **

*p*-value: differences between subgroups, ** chi-square test and *** Fisher exact test. A *p*-value < 0.05 is considered significant.

## Data Availability

The data used in the current study are available on reasonable request from the corresponding author.

## Abbreviations

Abbreviation	Meaning
AMD	adherence to the Mediterranean diet
BMI	body mass index
CG	control group
IG	intervention group
MD	Mediterranean diet
MDSS	Mediterranean diet serving score
MEDAS	Mediterranean diet adherence screener
NCD	non-communicable disease
SD	standard deviation

## Appendix A

**Table A1:** Usability Rating Questionnaire for e-12HR.

1. I found e-12HR easy to complete:
1 2 3 4 5
2. I found the questions of e-12HR understandable:
1 2 3 4 5
3. I found the feedback from e-12HR understandable (only for intervention group):
1 2 3 4 5
4. I would be willing to complete e-12HR again:
1 2 3 4 5
5. How much time to complete the app (per day):
<1 min/day Approx. 1 min/day Approx. 2 min/day Approx. 3 min/day Approx. 4 min/day 5 min/day or more

1: Strongly agree. 2: Agree. 3: Neither agree nor disagree. 4: Disagree. 5: Strongly disagree.

## References

[1] Yolcuoğlu İ.Z., Kızıltan G. (2022). Effect of Nutrition Education on Diet Quality, Sustainable Nutrition and Eating Behaviors among University Students. J. Am. Nutr. Assoc..

[2] Ferreira-Pêgo C., Rodrigues J., Costa A., Sousa B. (2020). Eating behavior: The influence of age, nutrition knowledge, and Mediterranean diet. Nutr. Health.

[3] Muñoz-Rodríguez J.R., Luna-Castro J., Ballesteros-Yáñez I., Pérez-Ortiz J.M., Gómez-Romero F.J., Redondo-Calvo F.J., Alguacil L.F., Castillo C.A. (2021). Influence of biomedical education on health and eating habits of university students in Spain. Nutrition.

[4] Deliens T., Clarys P., De Bourdeaudhuij I., Deforche B. (2014). Determinants of eating behaviour in university students: A qualitative study using focus group discussions. BMC Public Health.

[5] Widmer R.J., Flammer A.J., Lerman L.O., Lerman A. (2015). The Mediterranean diet, its components, and cardiovascular disease. Am. J. Med..

[6] WHO, FAO Diet, Nutrition, and the Prevention of Chronic Diseases. Report of a Joint WHO and FAO Expert Consulation. https://apps.who.int/iris/bitstream/handle/10665/42665/WHO_TRS_916.pdf;jsessionid=CFFE96E462ACDF9547B5F4A435B269FB?sequence=1.

[7] Gallus S., Bosetti C., La Vecchia C. (2004). Mediterranean diet and cancer risk. Eur. J. Cancer Prev..

[8] Salas-Salvadó J., Fernández-Ballart J., Ros E., Martínez-González M.A., Fitó M., Estruch R., Corella D., Fiol M., Gómez-Gracia E., Arós F. (2008). Effect of a Mediterranean diet supplemented with nuts on metabolic syndrome status: One-year results of the PREDIMED randomized trial. Arch. Intern. Med..

[9] Dernini S., Berry E.M., Serra-Majem L., La Vecchia C., Capone R., Medina F.X., Aranceta-Bartrina J., Belahsen R., Burlingame B., Calabrese G. (2017). Med Diet 4.0: The Mediterranean diet with four sustainable benefits. Public Health Nutr..

[10] Dernini S., Berry E.M. (2015). Mediterranean Diet: From a Healthy Diet to a Sustainable Dietary Pattern. Front. Nutr..

[11] Berry E.M., Dernini S., Burlingame B., Meybeck A., Conforti P. (2015). Food security and sustainability: Can one exist without the other?. Public Health Nutr..

[12] Burlingame B., Dernini S. (2012). Sustainable Diets and Biodiversity.

[13] Lacirignola C., Capone R. (2015). Mediterranean Food Consumption Patterns Diet, Environment, Society, Economy and Health.

[14] Burlingame B., Dernini S. (2011). Sustainable diets: The Mediterranean diet as an ex-ample. Public Health Nutr..

[15] Estruch R., Ros E., Salas-Salvadó J., Covas M.I., Corella D., Arós F., Gómez-Gracia E., Ruiz-Gutiérrez V., Fiol M., Lapetra J. (2018). Primary Prevention of Cardiovascular Disease with a Mediterranean Diet Supplemented with Extra-Virgin Olive Oil or Nuts. N. Engl. J. Med..

[16] Aranceta-Bartrina J., Arija-Val V.V., Maíz-Aldalur E., Martínez de Victoria-Muñoz E., Ortega-Anta R.M., Pérez-Rodrigo C., Quiles-Izquierdo J., Rodríguez-Martín A., Román-Viñas B., Salvador-Castell G. (2016). Dietary Guidelines for the Spanish population (SENC, December 2016); the new graphic icon of healthy food. Nutr. Hosp..

[17] García-Meseguer M.J., Burriel F.C., García C.V., Serrano-Urrea R. (2014). Adherence to Mediterranean diet in a Spanish university population. Appetite.

[18] Castro-Cuesta J.Y., Montoro-García S., Sánchez-Macarro M., Carmona-Martínez M., Espinoza-Marenco I.C., Pérez-Camacho A., Martínez-Pastor A., Abellán-Alemán J. (2022). Adherence to the Mediterranean diet in first-year university students and its association with lifestyle-related factors: A cross-sectional study. Hipertens. Riesgo Vasc..

[19] Telleria-Aramburu N., Arroyo-Izaga M. (2022). Risk factors of overweight/obesity-related lifestyles in university students: Results from the EHU12/24 study. Br. J. Nutr..

[20] Porto-Arias J.J., Lorenzo T., Lamas A., Regal P., Cardelle-Cobas A., Cepeda A. (2018). Food patterns and nutritional assessment in Galician university students. J. Physiol. Biochem..

[21] Bejar L.M. (2022). Weekend–Weekday Differences in Adherence to the Mediterranean Diet among Spanish University Students. Nutrients.

[22] Martinez-Lacoba R., Pardo-Garcia I., Amo-Saus E., Escribano-Sotos F. (2018). Socioeconomic, demographic and lifestyle-related factors associated with unhealthy diet: A cross-sectional study of university students. BMC Public Health.

[23] Moreno-Gómez C., Romaguera-Bosch D., Tauler-Riera P., Bennasar-Veny M., Pericas-Beltran J., Martinez-Andreu S., Aguilo-Pons A. (2012). Clustering of lifestyle factors in Spanish university students: The relationship between smoking, alcohol consumption, physical activity and diet quality. Public Health Nutr..

[24] Tárraga-López P.J., Tárraga-Marcos A., Panisello J.M., Herencia-Carbayo J.A., Tárraga-Marcos M.L., López-Gil J.F. (2022). Physical activity and its association with Mediterranean diet patterns among Spanish university students. Nutr. Hosp..

[25] Morris M.A., Wilkins E.L., Galazoula M., Clark S.D., Birkin M. (2020). Assessing diet in a university student population: A longitudinal food card transaction data approach. Br. J. Nutr..

[26] Štefan L., Čule M., Milinović I., Sporiš G., Juranko D. (2017). The relationship between adherence to the Mediterranean diet and body composition in Croatian university students. Eur. J. Integr. Med..

[27] Li Y., Roswall N., Ström P., Sandin S., Adami H.O., Weiderpass E. (2015). Mediterranean and Nordic diet scores and long-term changes in body weight and waist circumference: Results from a large cohort study. Br. J. Nutr..

[28] Grillone L., Castriotta L., Antinolfi F., Righini M., Brusaferro S., Parpinel M. (2018). University students’ Mediterranean diet adherence in North East of Italy: A pilot study, 2018. Eur. J. Public Health.

[29] de-Mateo-Silleras B., Camina-Martín M.A., Cartujo-Redondo A., Carreño-Enciso L., de-la-Cruz-Marcos S., Redondo-Del-Río P. (2019). Health Perception According to the Lifestyle of University Students. J. Community Health.

[30] Béjar L.M., García-Perea M.D., Reyes Ó.A., Vázquez-Limón E. (2019). Relative Validity of a Method Based on a Smartphone App (Electronic 12-Hour Dietary Recall) to Estimate Habitual Dietary Intake in Adults. JMIR mHealth uHealth.

[31] Béjar L.M., García-Perea M.D., Mesa-Rodríguez P. (2022). Evaluation of an Application for Mobile Telephones (e-12HR) to Increase Adherence to the Mediterranean Diet in University Students: A Controlled, Randomized and Multicentric Study. Nutrients.

[32] Monteagudo C., Mariscal-Arcas M., Rivas A., Lorenzo-Tovar M.L., Tur J.A., Olea-Serrano F. (2015). Proposal of a Mediterranean Diet Serving Score. PLoS ONE.

[33] Recio-Rodriguez J.I., Agudo-Conde C., Martin-Cantera C., González-Viejo M.N., Fernandez-Alonso M.D., Arietaleanizbeaskoa M.S., Schmolling-Guinovart Y., Maderuelo-Fernandez J.A., Rodriguez-Sanchez E., Gomez-Marcos M.A. (2016). Short-Term Effectiveness of a Mobile Phone App for Increasing Physical Activity and Adherence to the Mediterranean Diet in Primary Care: A Randomized Controlled Trial (EVIDENT II Study). J. Med. Internet Res..

[34] Recio-Rodriguez J.I., Agudo Conde C., Calvo-Aponte M.J., Gonzalez-Viejo N., Fernandez-Alonso C., Mendizabal-Gallastegui N., Rodriguez-Martin B., Maderuelo-Fernandez J.A., Rodriguez-Sanchez E., Gomez-Marcos M.A. (2018). The Effectiveness of a Smartphone Application on Modifying the Intakes of Macro and Micronutrients in Primary Care: A Randomized Controlled Trial. The EVIDENT II Study. Nutrients.

[35] Alonso-Domínguez R., García-Ortiz L., Patino-Alonso M.C., Sánchez-Aguadero N., Gómez-Marcos M.A., Recio-Rodríguez J.I. (2019). Effectiveness of A Multifactorial Intervention in Increasing Adherence to the Mediterranean Diet among Patients with Diabetes Mellitus Type 2: A Controlled and Randomized Study (EMID Study). Nutrients.

[36] Gonzalez-Ramirez M., Sanchez-Carrera R., Cejudo-Lopez A., Lozano-Navarrete M., Salamero Sánchez-Gabriel E., Torres-Bengoa M.A., Segura-Balbuena M., Sanchez-Cordero M.J., Barroso-Vazquez M., Perez-Barba F.J. (2022). Short-Term Pilot Study to Evaluate the Impact of SalBi Educa Nutrition App in Macronutrients Intake and Adherence to the Mediterranean Diet: Randomized Controlled Trial. Nutrients.

[37] Vasiloglou M.F., Christodoulidis S., Reber E., Stathopoulou T., Lu Y., Stanga Z., Mougiakakou S. (2020). What Healthcare Professionals Think of “Nutrition & Diet” Apps: An International Survey. Nutrients.

[38] Béjar L.M., Sharp B.N., García-Perea M.D. (2016). The e-EPIDEMIOLOGY Mobile Phone App for Dietary Intake Assessment: Comparison with a Food Frequency Questionnaire. JMIR Res. Protoc..

[39] Béjar L.M., Vázquez-Limón E. (2017). Is there any alternative to traditional food frequency questionnaire for evaluating habitual dietary intake?. Nutr. Hosp..

[40] Béjar L.M. (2017). First evaluation steps of a new method for dietary intake estimation regarding a list of key food groups in adults and in different sociodemographic and health-related behaviour strata. Public Health Nutr..

[41] Béjar L.M., Reyes Ó.A., García-Perea M.D. (2018). Electronic 12-Hour Dietary Recall (e-12HR): Comparison of a Mobile Phone App for Dietary Intake Assessment with a Food Frequency Questionnaire and Four Dietary Records. JMIR mHealth uHealth.

[42] Prochaska J.O., Velicer W.F. (1997). The transtheoretical model of health behavior change. Am. J. Health Promot..

[43] Willett W. (2013). Nutritional Epidemiology.

[44] Forster H., Fallaize R., Gallagher C., O’Donovan C.B., Woolhead C., Walsh M.C., Macready A.L., Lovegrove J.A., Mathers J.C., Gibney M.J. (2014). Online dietary intake estimation: The Food4Me food frequency questionnaire. J. Med. Internet Res..

[45] Rutishauser I.H. (2005). Dietary intake measurements. Public Health Nutr..

[46] Tucker K.L., Smith C.E., Lai C.Q., Ordovas J.M. (2013). Quantifying diet for nutrigenomic studies. Annu. Rev. Nutr..

[47] Gibson R. (2005). Principles of Nutritional Assessment.

[48] Martín-Moreno J.M., Gorgojo L. (2007). Assessment of dietary intake at the population level through individual questionnaires: Methodological shadows and lights. Rev. Esp. Salud Publica.

[49] Dhurandhar N.V., Schoeller D., Brown A.W., Heymsfield S.B., Thomas D., Sørensen T.I., Speakman J.R., Jeansonne M., Allison D.B. (2015). Energy Balance MeasurementWorking Group. Energy balance measurement: When something is not better than nothing. Int. J. Obes..

